# Effect of innovative vs. usual care physical therapy in subacute rehabilitation after stroke. A multicenter randomized controlled trial

**DOI:** 10.3389/fresc.2022.987601

**Published:** 2022-09-19

**Authors:** Marianne Sivertsen, Ellen Christin Arntzen, Karl Bjørnar Alstadhaug, Britt Normann

**Affiliations:** ^1^ Department of Health and Care Sciences, UiT The Arctic University of Norway, Tromsoe, Norway; ^2^ Department of Medicine, Nordland Hospital Trust, Bodoe, Norway; ^3^ Faculty of Nursing and Health Sciences, Nord University, Bodoe, Norway; ^4^ Department of Clinical Medicine, UiT The Arctic University of Norway, Tromsoe, Norway

**Keywords:** physical therapy, stroke, rehabilitation, trunk control, balance, gait, physical activity, health related quality of life

## Abstract

**Background:**

Research on stroke rehabilitation often addresses common difficulties such as gait, balance or physical activity separately, a fragmentation contrasting the complexity in clinical practice. Interventions aiming for recovery are needed. The purpose of this study was to investigate effects of a comprehensive low-cost physical therapy intervention, I-CoreDIST, vs. usual care on postural control, balance, physical activity, gait and health related quality of life during the first 12 weeks post-stroke.

**Methods:**

This prospective, assessor-masked randomized controlled trial included 60 participants from two stroke units in Norway. Participants, who were randomized to I-CoreDIST (*n* = 29) or usual care physical therapy (*n* = 31), received 5 sessions/week when in-patients or 3 sessions/week as out-patients. Primary outcomes were the Trunk Impairment Scale-modified Norwegian version (TISmodNV) and activity monitoring (ActiGraphsWgt3X-BT). Secondary outcomes were the Postural Assessment Scale for Stroke, MiniBesTEST, 10-meter walk test, 2-minute walk test, force-platform measurements and EQ5D-3L. Stroke specific quality of life scale was administered at 12 weeks. Linear regression and non-parametric tests were used for statistical analysis.

**Results:**

Five participants were excluded and seven lost to follow-up, leaving 48 participants in the intention-to-treat analysis. There were no significant between-group effects for primary outcomes: TIS-modNV (*p* = 0,857); daily average minutes of sedative (*p* = 0.662), light (*p* = 0.544) or moderate activity (*p* = 0.239) and steps (*p* = 0.288), or secondary outcomes at 12 weeks except for significant improvements on EQ5D-3L in the usual care group. Within-group changes were significant for all outcomes in both groups except for activity levels that were low, EQ5D-3L favoring the usual care group, and force-platform data favoring the intervention group.

**Conclusions:**

Physical therapy treatment with I-CoreDIST improved postural control, balance, physical activity and gait during the first 12 weeks after a stroke but is not superior to usual care.

## Introduction

Stroke is a common cause of physical and cognitive disabilities. It is associated with lower levels of health-related quality of life (HRQOL) (1) and low levels of physical activity both during in-patient rehabilitation (2, 3) and in the long term (4, 5). Physical therapy is integral to the rehabilitation chain after a stroke, and is effective in reducing the burden of disability (6, 7). Strong evidence exists to support that increased dose and intensity of physical therapy increase functional gains (6). Recommendations, however, are often not achieved.

Research on stroke rehabilitation often addresses either gait, balance or upper limb function or specific treatments targeting single impairments (8). This fragmentation in research is in contrast to the complexity encountered by physical therapists in clinical stroke rehabilitation (9, 10), where the patients' movement problem often constitutes a combination of impairments and their mutual influence on each other. The main aims of physical therapy after a stroke are to improve walking, balance and functional movement (6), for which trunk control is a prerequisite (11–13). Reduced trunk control is common after a stroke and often persists into the sub-acute and chronic phases (12, 13). Such dysfunction is associated with poor functional mobility, reduced independence in activities of daily living and increased risk of falls (13–15). Recent reviews have concluded that there is evidence to support that trunk control, sitting and standing balance and mobility may significantly improve following trunk training after a stroke (13, 16–18). Findings support intensive rehabilitation treatment targeting trunk control to regain mobility and gait early after a stroke (14). The examined effect of trunk training is often in addition to usual care, thus separating the training of trunk control from the training of functional tasks, balance and gait. In daily activities these are inextricably linked, for example through the fine adjusted timing of anticipatory postural adjustments, that occur prior to the center of mass displacements associated with movements (19). The timing and symmetry of anticipatory postural adjustments are often affected after a stroke (20). There is a need to investigate if integrating trunk training and usual care could lead to greater functional gains.

New interventions in stroke rehabilitation should comprise clearly defined evidence (Langhorne 2009) and science-based methods (Nielsen 2015), and should aim to enhance recovery as opposed to compensatory strategies (21, 22). I-CoreDIST^1^ (Table 1) is a comprehensive, innovative rehabilitation method where activation of core muscles is enhanced and integral to all exercises without compromising focus on functional tasks or intensity. We support Kibler's (23) definition of core stability as “the ability to control the position and motion of the trunk over the pelvis and leg to allow optimum production, transfer and control of force and motion to the terminal segment in integrated kinetic chain activities”, (p. 190). This view incorporates an extended perspective of core muscles as all muscles on the trunk and those attached to the trunk, thus including muscles on the shoulder and hip girdle. The novelty of this approach lies within its integration of core muscle activation into exercises that incorporate functional activities, muscle strength, active muscle lengthening, upper limb function, gait and endurance. The structured assessment, clinical reasoning aids and the variation of exercises ensures individual tailoring and specificity. I-CoreDIST is designed to follow the patient through the course of rehabilitation, thus addressing fragmentation of care delivery and lack of continuity between care centers, a recognized barrier to recovery in stroke rehabilitation (9, 24, 25). The implementation of I-CoreDIST in the sub-acute stage after a stroke has successfully been explored in a non-controlled pilot study that demonstrated significant improvements in balance, postural control, walking-speed and -distance from baseline to 4 and 12 weeks (26).

**Table 1:** Outline of the I-core DIST intervention.

Main Features	Assessment	Exercises
Common features in all exercises are enhancement of dynamic trunk stability and functional movements, combined with the following: Optimized alignment and adaptation to the base of support and often using an unstable reference point for the trunk (therapeutic ball) or the distal body parts. Enhanced somatosensory integration of hands, feet and face, including reduced influence of vision to enhance somatosensory integration.Proximal stability prior to selective task-oriented movement of limbs, head, eyes. Inclusion of dual tasks (motor/motor and motor/cognitive) in exercises and activities such as walking indoors, out-doors and climbing stairs. Specific hands-on interactions or other adaptations to optimize alignment and neuromuscular recruitment. Exercises combining core activation and increase in heart rate: in lying, sitting, standing and walking.	• History • General function • Specific assessment • Exploration of possibilities for change • Conclusions • Goals • Clinical reasoning charts for assistance • Example of clinical reasoning chart: 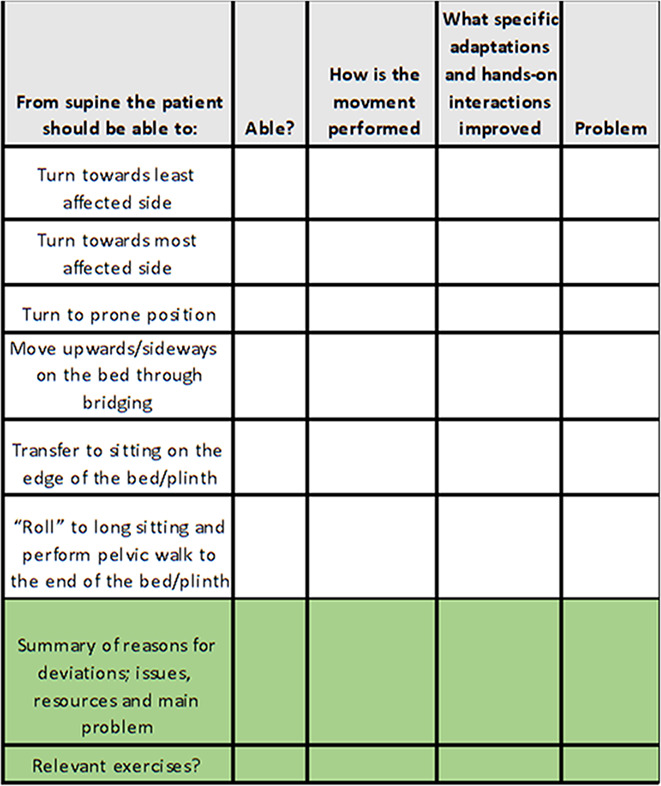	44 exercises, each with five levels of difficulty and choices of starting positions: • Supine • Side-lying • Prone • Sitting • Standing • Stepping and walking. All individual exercises have been assigned a color, indicating the main aims: 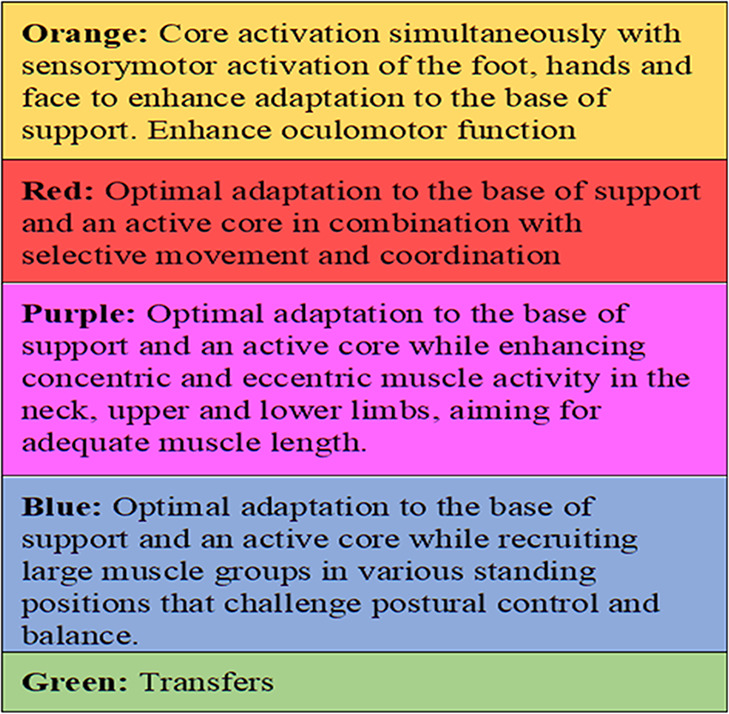

The purpose of this study was to investigate the effect of I-CoreDIST when implemented in sub-acute, post-stroke physical therapy by addressing the following research question: Is physical therapy with I-CoreDIST better at improving postural control, levels of physical activity, balance, gait and HRQOL than usual care physical therapy when implemented during the first 12 weeks after a stroke.

## Materials and methods

### Trial design

This assessor-blinded, two arm parallel group, randomized controlled trial (RCT) was registered at ClinicalTrials.gov (ClinicalTrials.gov Identifier: NCT04069767) prior to inclusion of participants. The study adhered to the CONSORT guidelines and to guidelines for data protection set by the involved institutions.

### Ethics

The study was approved by the Regional Committee of Medical and Health Research Ethics (REK North: 2017/1961) and complies with the Declaration of Helsinki. All participants provided written informed consent prior to inclusion. The funders played no role in the design, conduct or reporting of this study.

### Context of the study

The study was conducted in collaboration with two hospitals in two regions of Norway, two rehabilitation units and six surrounding municipalities. Participants were recruited at the hospitals stroke units where they underwent baseline testing prior to discharge and a follow-up assessment after 12 weeks. Inclusion started in September 2019 and ended in December 2021. Due to lockdown and subsequent restrictions related to the Covid-19 pandemic, inclusion and physiotherapy treatment for already included participants were stopped between March and June 2020.

### Participants

Eligible participants, aged 18–85, had to be admitted to one of the two stroke units with a confirmed new stroke, have a premorbid modified Ranking Scale (mRS) of 0–3, be able to sit for 10 s at baseline testing, and to have a Trunk Impairment Scale-modified Norwegian version (TIS-modNv) score of <15. Exclusion criteria were inability to cooperate in physical therapy, ongoing substance abuse, severe disease, known dementia or other mental or cognitive disability preventing participation in physical therapy. After inclusion a baseline-assessment, evaluating trunk control, balance and gait along with self-administered questionnaire on health-related quality of life, was administered.

### Randomization

After baseline assessment, the participants were randomly assigned to one of two trial arms, A and B, in a 1:1 ratio. Randomization was stratified into two groups based on functional disability at baseline defined by mRS < 4 or ≥4 to minimize selection bias and to preserve homogeneity between arms. A digital solution, RedCap (Research Electronic Data Capture) tools hosted at the Northern Norway Regional Health Authority was used for randomization and data collection. Randomization was performed by an investigator, not connected to assessment or treatment of the patients, who informed the relevant physical therapist at rehabilitation units and/or municipalities of group allocation. The participants and the outcome assessors were blinded to group allocation.

### Interventions, I-CoreDIST and usual care

**Figure 1:**
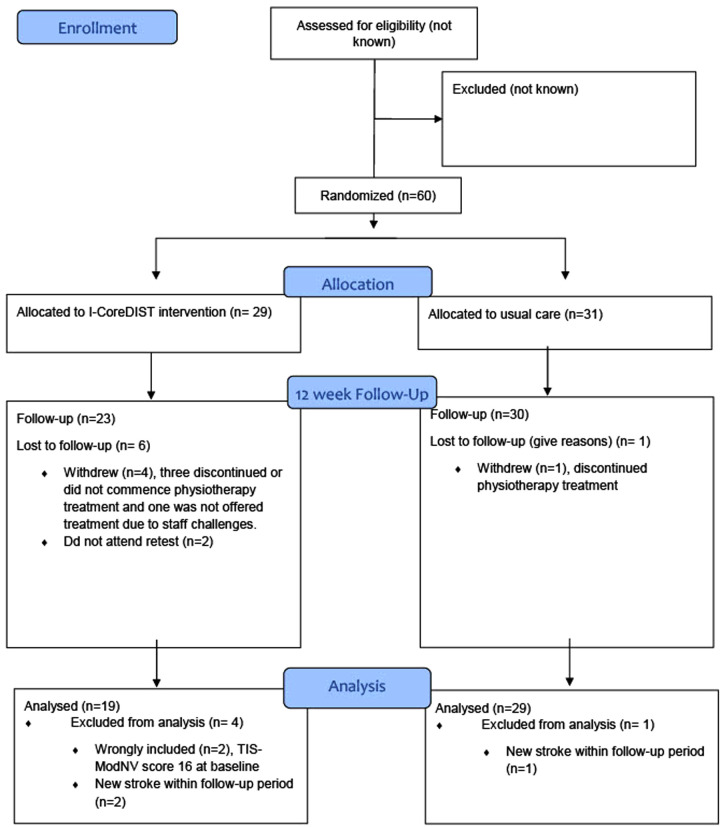
The flow of patients through the study.

The flow of patients through the study is summarized in Figure 1. The intervention period commenced after discharge from the stroke unit and lasted through the patient's individual rehabilitation course for 12 weeks. Time of and destination at discharge were not affected by participation in the study. Each physical therapy session lasted 60 min and was performed 5–6 days/per week if in a rehabilitation unit, and 3 sessions/week if in home based or outpatient treatment. Both groups received equal doses of physical therapy. Written reports followed the patient throughout the rehabilitation chain along with medical and multidisciplinary care as usual. Registrations of frequency and content of I-CoreDIST and usual care sessions were recorded for 12 weeks by the physical therapists.

#### I-CoreDIST

**Figure 2:**
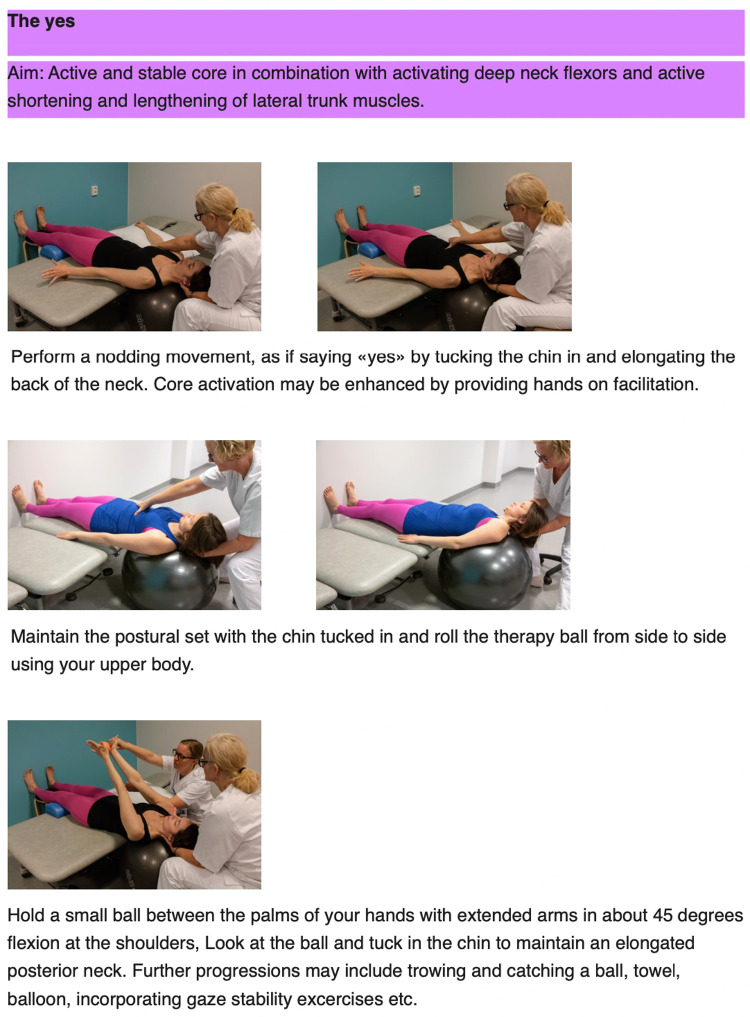
Example of exercise aiming for optimal adaptation to the base of support, an active core as well as enhancement of concentric and eccentric mucle activity in the neck.

**Figure 3:**
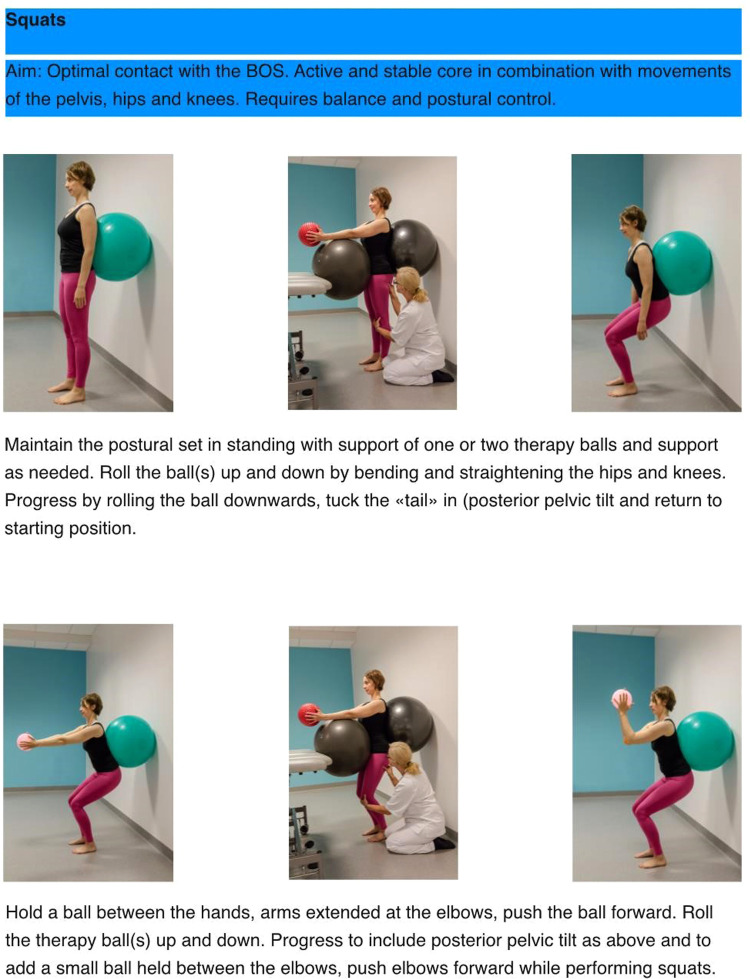
Example of exercise aiming for optimal adaptation to the base of support, an active core, activity in large muscle groups in a standing position while challenging postural control and balance.

**Figure 4:**
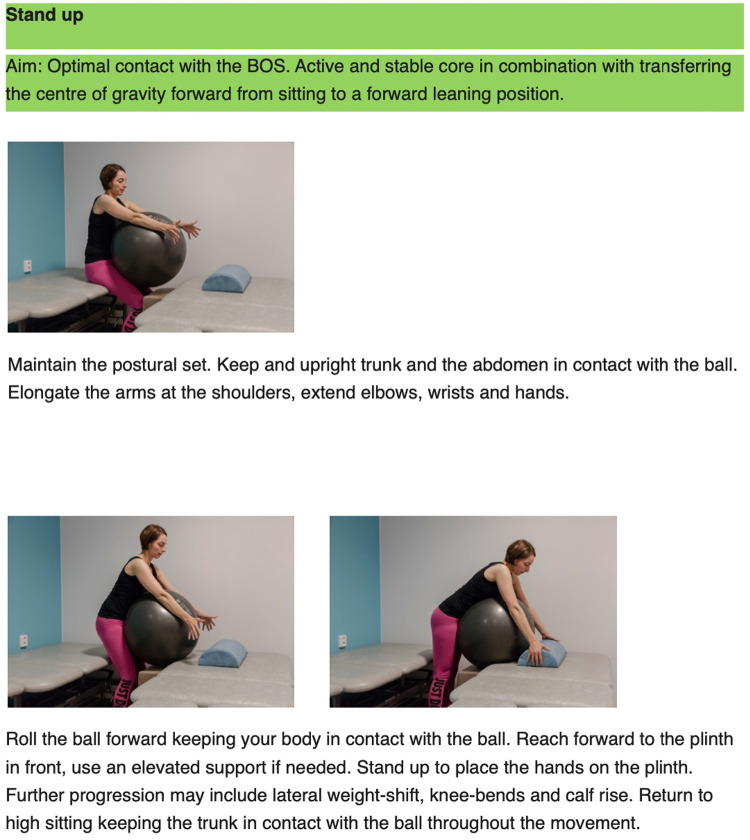
Example of exercise that aim for optimal adaptation to the base of support, an active core while practicing transferring the centre of gravity forward as in a sit to stand transfer.

The principles behind the I-CoreDIST intervention is outlined in Table 1. In I-CoreDIST structured core muscle activation is actively incorporated into exercises that simultaneously demand muscle strength, active muscle lengthening and endurance. These exercises specifically aim to improve, balance, gait, transfers upper limb function and functional activities, thus enhancing the training of the specific aspects of trunk function needed in everyday activities. The intervention started with an assessment to identify the patient's movement problems, supported by clinical reasoning charts, and contains 44 exercises, each with five levels of difficulty to allow for specificity and individualization. All physical therapists who treated participants in the I-CoreDIST group received 45 h of training prior to commencement of the study, one follow-up day during inclusion, and an educational package containing (1) the theoretical rationale behind the approach, (2) assessment and clinical reasoning charts and (3) images and descriptions of all exercises (Figures 2–4).

#### Usual care

There were no guidelines regarding the content of physical therapy, each individual therapist made treatment choices according to existing guidelines and what was usually offered to this patient group in that particular institution or municipality. The content of usual care in clinical practice in Norway is highly variable and poorly documented. Approaches towards stroke rehabilitation vary between the different schools of physiotherapy and traditions within institutions and municipalities. The Norwegian guidelines for treatment and rehabilitation after a stroke provide only general advice on including; intensive task related training containing a strength component for patients with paresis, training of transfers, gait and cardiovascular fitness, bilateral or constraint induced arm training (27). Specific training of trunk control is not a part of the recommendation for rehabilitation of sensorimotor disturbances after a stroke (27), but is part of the treatment tradition in some institutions.

### Outcome measures

The primary outcomes were trunk control, evaluated by TIS-modNV and physical activity, measured by an accelerometer and quantified into sedentary time, time in light, moderate and vigorous activity and number of steps. TIS-modNV is a 0–16-point scale, for which the ability to sit without support for 10 s is a prerequisite. It is considered a valuable tool for evaluation of trunk control and The scale has been proven reliable (ICC = 0.85) and valid for the stroke population (28), is sensitive, and do not have a ceiling effect.The minimal detectable change (MDC) is 2.9 points (28). ActiGraph Wgt3X-BT (ActiGraph, LCC, Pensacola, United States) is a 3-axis accelerometer used to record physical activity. It has been proven reliable in an adult population (29) and valid (ICC = 0.70) for use in the stroke population (30). Levels of physical activities are reflective of recovery of the activity limitation often experienced by stroke patients (31). The participants were instructed to wear the activity monitor in a waistband 24 h/day for seven consecutive days, after both baseline testing and the 12-week follow-up assessment. The participants were instructed to remove the device during showers/baths only. The devices were initialized and data were downloaded using ActiLife Software (ActiGraph, LCC, Pensacola, United States). Data were collected at a frequency of 100 Hz.

Secondary outcomes were postural control, balance, gait speed and distance, and HRQOL. We used the Swedish Postural Assessment Scale for stroke -Norwegian Version (SwePASS-NV) to measure postural control and the ability to maintain equilibrium during positional changes. It is sensitive for assessment of postural control after a stroke, and has excellent validity (*α* = 0.99, *p* < 0.001) (32), and reliability (ICC ≥ 0.99) (33). The scale ranges from 0 to 36 and has a ceiling, but no floor effect. The MDC in subacute stroke is 2.2 points (34). MiniBESTest was used to measure pro-and reactive balance in standing and walking on a scale from 0 to 28. It has a floor effect, as participants must be able to stand without support. The Norwegian version has shown good reliability (ICC = 0.95) and validity (35). The MDC for MiniBESTest is 3.2 points. In addition, the minimal clinically important difference (MCID) for detecting small changes is 4 points and five points for detecting substantial changes (36). Stability during quiet stance was assessed by calculating sway amplitude using AMTI AccuGait Optimized™ (Advanced Mechanical technology, Inc., Watertown, United States) multi-axis force plate system. Data on center of pressure (COP) displacements in cm were collected for 30 s with a frequency of 50 Hz (37) in the domains of eyes open and eyes closed and root mean square (RMS) values of the COP displacements were calculated. Reliability has been established for measuring COP displacements during quiet stance in the anteroposterior (AP) (ICC = 0.77) and mediolateral (ML) (ICC = 0.74) directions in a stroke population (38). Participants who were able to walk with or without an aid performed: (1) 10-Meter Walk Test (10 MWT), measuring walking speed (meters/s) at preferred and fast paces, reliable (ICC = 0.76) and valid for use in the stroke population (39, 40). MCID for 10 MWT preferred pace is 0.16 m/s (41) and 0.13 m/s for the 10 MWT fast pace (42) and (2) The 2-Minute Walk Test (2 MWT), measuring the total distance walked in two minutes, conducted on a 20 m walkway, also reliable (ICC = 0.85) for the stroke population (43). For non-ambulant participants, 0 meter/s was recorded at baseline or 12 weeks. HRQOL was reported using EQ-5D-3L and the stroke specific quality of life scale (SSQOL). EQ-5D-3L is a questionnaire used to assess self-perceived HRQOL, comprising five dimensions: mobility, self-care, usual activities, pain/discomfort and anxiety/depression, each with three levels of response, and a VAS scale (0–100) recording perceived health (44). EQ5D-3L has been proven reliable and valid for use in a stroke population (45, 46). SSQOL assesses health-related quality of life specific for stroke survivors. It is a 49-item questionnaire, addressing 12 domains: self-care, vision, language, mobility, work/productivity, upper extremity function, thinking, personality, mood, family roles, social roles and energy (47). The Norwegian translation has shown excellent reliability (ICC = 0.97) and validity (48). SSQOL was administered only at 12 weeks retest as it was not considered appropriate in the acute stage.

### Sample size

Sample size was calculated based on the mean and standard deviation of TIS-modNV-scores from a preceding pilot study (26). A difference of 0.67 standard deviation (SD) (1.93 points) between the intervention and the control group was considered clinically relevant. Thirty-seven individuals in each group were required to obtain an 80% chance to detect a difference of 1.93 points on TIS-modNV between the groups with a significance level of 0.05 (alpha) on two-sided tests.

### Data analysis

Prior to statistical analysis the COP data were filtered using a fourth order Butterworth filter applied at 10 Hz (49) using BalanceClinic software (AMTI). The raw COP-data were imported to MatLab (Mathworks, Natick, MA, United States) where average RMS-values of COP-displacements in the AP (COPy) and ML (COPx) planes were calculated using the formula $\mathrm{RMS}\;\mathrm{AP}=\sqrt{\frac{1}{n}}\left(y_{1}^{2}+y_{2}^{2}+y_{n}^{2}\right)$, and $\mathrm{RMS}\;\mathrm{ML}=\sqrt{\frac{1}{n}}\left(x_{1}^{2}+x_{2}^{2}+x_{n}^{2}\right)$. Raw activity data were converted into daily average minutes of sedative time, light, moderate and vigorous activity using the ActiLife Software (ActiGraph, LCC, Pensacola, United States). Data were downloaded for all days, but day 1 and 8 were excluded due to differences in starting time. EQ5D profiles were summarized by calculating index values for each respondent (50). We utilized the value set from Denmark (51) as there is no available sets for Norway. This value set has previously been utilized in a Norwegian stroke population (52). Index values were also calculated for the SSQOL-data, converting scores from the 49 individual items into average scores for the 12 domains. Missing data were handled using person mean imputation and replaced by the domain average if one missing in a three-question domain or two missing in a five/six question domain. Forms were discarded if more than five missing items.

### Statistical analysis

We performed an intention-to-treat analysis. Continuous variables are presented as means and standard deviations (SD) or median and interquartile range (IQR) depending on normality distribution. Categorical variables are presented as numbers and percentages. A multiple linear regression model was used to test if group allocation significantly predicted 12-week retest score when adjusting for baseline scores. If the data violated the assumptions for linear regression analysis, we performed a natural log transformation or used a Mann-Whitney U test for between-group differences. Within-group differences were calculated using paired samples t-test given a normal distribution of data and Wilcoxon signed rank test if not. Significance level was set at *p* < 0.05. All analyses were carried out using IBM SPSS (Statistics version 27 SPSS INC., Chicago IL).

## Results

A total of 60 participants were recruited between September 2019 and September 2021. Baseline characteristics are outlined in Table 2. Twentynine participants were randomized to the intervention group (I-CoreDIST) and 31 to the usual care group (Figure 1). The groups did not significantly differ in baseline characteristics, but there was a trend towards higher mean age (*p* = 0.17), lower premorbid levels of function (mRS) (*p* = 0.12) and higher scores for stroke severity on the NIH Stroke Scale (NIHSS) (*p* = 0.22) in the intervention group. The intervention group also had a higher rate of bilateral strokes, while the control group had a higher rate of hemorrhagic strokes. In the intervention group, six participants were lost to follow-up and another four were excluded from analysis. In the usual care group, one was lost to follow-up and one was excluded from analysis (Figure 1).

**Table 2:** Demographic data.

Baseline characteristics	Intervention group (*n* = 25)	Control group (*n* = 30)	*p*
**Age: mean (SD)**	72.96 (10.41)	69.32 (10.63)	0.17
**Gender**			
Male, *n* (%)	12 (48)	23 (76.66)	
Female, *n* (%)	13 (52)	7 (23.33)	
Cohabiting, *n* (%)	17 (68)	21 (70)	
**Premorbid mRS mean (SD) (inclusion criteria: mRS < 4)**	0.83 (1.09)	0.46 (0.15)	0.12
**Type of stroke**			
Infarction, *n* (%)	24 (96)	26 (86.66)	
Hemmorage, *n* (%)	1 (4)	4 (13.33)	
**Stroke location**			
Right hemisphere, *n* (%)	11 (44)	15 (50)	
Left hemisphere, *n* (%)	10 (40	14 (46.7)	
Bilateral, *n* (%)	4 (16)	1 (3.3)	
**NIHSS score at admission: mean (SD)**	5.04 (1.08)	3.64 (0.58)	0.22
**Barthel Index admission: mean (SD)**	82.29 (26.33)	81.07 (21.14)	0.85
**Previous stroke, *n* (%)**	7 (28)	6 (20)	

We used a multiple linear regression model for TIS-modNV, SwePASS-NV, MiniBesTEST, 10 MWT preferred and fast paces, activity data and 2 MWT and EQ5D-3L-scores (Table 3). There were some missing activity data at baseline as four monitors were not returned. In addition, three were excluded from analysis due to faulty monitors or a lack of registered activity in bouts exceeding that presumed to be inactivity. At retest, 15 participants did not attend or did not return the monitor, one was excluded due to little wear-time. Data in the categories of average minutes of moderate activity and average number of steps per day were skewed, thus natural log transformation were performed. The fitted regression model was a poor fit for the force platform data even after log transformation and as a result non-parametric tests were used to determine between-group differences.

**Table 3:** Regression model.

Outcome measure	ANOVA				Coefficients				
	*R* ^2^	*F*(2,45)	*p*		*B*	95% CI	*β*	*t*	*p*
**Primary**									
TIS-modNv	0.37	39.64	<0.001	Constant	4.81	2.39, 7.24		3.99	<0.001
				Group allocation	−0.14	−1.38, 1.16	−0.02	−0.18	0.86
				Baseline score	0.75	0.58, 0.92	0.80	8.90	<0.001
**Activity data**									
Sed mins/day	0.21	4.43	0.02	Constant	672.02	281.60, 1062.44		3.50	0.001
				Group allocation	38.90	−25.47, 103.27	0.19	1.23	0.23
				Baseline score	0.39	0.09, 0.69	0.41	2.66	0.01
Light mins/day	0.21	4.42	0.02	Constant	206.11	90.47, 321.75		3.63	0.001
				Group allocation	−43.66	−104.65, 17.33	−0.23	−1.46	0.16
				Baseline score	0.37	0.08, 0.67	0.40	2.56	0.02
Mod mins/day*	0.33	7.33	0.003	Constant	0.19	−1.18, 1.57		0.29	0.78
				Group allocation	0.59	−0.18, 1.35	0.24	1.57	0.13
				Baseline score	0.56	0.25, 0.88	0.55	3.67	0.001
Steps/day*	0.21	4.47	0.02	Constant	4.50	2.19, 6.81		3.96	<0.001
				Group allocation	0.03	−0.48, 0.55	0.20	0.13	0.89
				Baseline score	0.44	0.14, 0.74	0.46	2.96	0.01
**Secondary**									
SwePASS-NV	0.60	33.19	<0.001	Constant	18.31	14.20, 22.45		8.98	<0.001
				Group allocation	−0.33	−1.62, 0.96	−0.05	−0.52	0.61
				Baseline score	0.5	0.38, 0.62	0.78	8.13	<0.001
MiniBesTEST	0.51	22.95	<0.001	Constant	9.87	4.80, 14.95		3.92	<0.001
				Group allocation	1.42	−1.34, 4.18	0.11	1.03	0.31
				Baseline score	0.52	0.36, 0.68	0.70	6.69	<0.001
10 MWT (m/s)	0.50	22.40	<0.001	Constant	0.56	0.34, 0.78		5.17	<0.001
				Group allocation	0.07	−0.05, 0.13	0.13	1.22	0.23
				Baseline score	0.43	0.29, 0.56	0.68	6.45	<0.001
10 MWT fast (m/s)	0.49	21.42	<0.001	Constant	0.58	0.21, 0.96		3.12	0.003
				Group allocation	0.16	−0.04, 0.37	0.17	1.62	0.11
				Baseline score	0.51	0.34, 0.67	0.67	6.28	<0.001
2 MWT (m)	0.53	25.26	<0.001	Constant	71.120	34.20, 108.04		3.88	<0.001
				Group allocation	12.282	−8.37, 32.93	0.12	1.20	0.24
				Baseline score	0.501	0.35, 0.69	0.71	6.85	<0.001
EQ5D index	0.55	21.08	<0.001	Constant	0.268	0.75, 0.46		2.83	0.01
				Group allocation	0.154	0.29, 0.60	0.37	3.20	0.003
				Baseline score	0.442	0.06, 0.25	0.66	5.74	<0.001

* Natural log transformations were performed.

Group allocation was not a significant predictor of 12-week retest score when adjusted for baseline differences for the primary outcomes TIS-modNV (*p* = 0.857), or for the activity data across all categories: Sedative minutes/day (*p* = 0.228), minutes of light activity/day (*p* = 0.155), minutes of moderate activity/day (*p* = 0.127), average number of steps/day (*p* = 0.887) (Table 3). Paired samples t-tests revealed significant within-group changes for TIS-modNV (*p* < 0.001) in both groups (Table 4) and Wilcoxons signed rank test showed significant within group changes in favor of the usual care group in the categories “minutes of moderate activity” per day (*p* = 0.005) and “average number of steps/day” (*p* = 0.042) for the activity data. There was a trend towards lower *p*-values for the intervention group regarding reduction in sedative minutes/day and increase in minutes of light activity/day (Table 4).

**Table 4:** Within-group changes.

Primary outcome measures					
Outcome	Group	Baseline	12-week retest	Change	Paired samples *t*-test
		Mean (SD)	Mean (SD)	Mean difference 95%CI	*p*
TIS-Nv score	Intervention	7.37 (3.53)	10.21 (3.29)	2.84 1.85, 3.84	<0.001
	Usual care	7.79 (3.87)	10.41 (3.63)	2.62 1.69, 3.55	<0.001
**Outcome**	**Group**	**Baseline**	**12-week retest**	**Change**	**Wilcoxon signed rank test**
**Activity data**		**Median [IQR]**	**Median [IQR]**	**Median difference**	***Z*, *p***
Sedative mins/day	Intervention	1,268 [152]	1,241 [189]	−27	−1.41, 0.16
	Usual care	1,270 [164]	1,263 [104]	−7	−0.83, 0.41
Light act mins/day	Intervention	163 [132]	199 [189]	36	−1.41, 0.16
	Usual care	157 [163]	164 [120]	7	−0.57, 0.57
Mod acti mins/day	Intervention	1 [13]	2.5 [8]	1,5	−0.27, 0.79
	Usual care	3 [5]	8 [26]	5	−2.84, 0.005
Vig act mins/day	Intervention	0 [0]	0 [0]	0	0, 1.0
	Usual care	0 [0]	0 [0]	0	0, 1.0
Steps/day	Intervention	1,723 [2,718]	2,099 [2,880]	376	−1.35, 0.18
	Usual care	1,575 [2,301]	3,327 [3,170]	1752	−2.03, 0.04
**Secondary outcome measures**					
**Outcome**	**Group**	**Baseline**	**12-week retest**	**Change**	**Paired samples *t*-test**
		**Mean (SD)**	**Mean (SD)**	**Mean difference 95%CI**	***p***
MiniBesTEST score	Intervention	13.47 (9.48)	18.32 (6.57)	4.84 2.22, 7.46	<0.001
	Usual care	13.65 (8.29)	19.83 (6.43)	6.17 3.63, 8.71	<0.001
10 mwt, (m/s)	Intervention	0.72 (0.47)	0.94 (0.30)	0.22 0.07, 0.37	0.007
	Usual care	0.80 (0.44)	1.05 (0.26)	0.24 0.12, 0.37	<0.001
10 mwt fast (m/s)	Intervention	1.05 (0.70)	1.28 (0.44)	0.23 −0.01, 0.47	0.06
	Usual care	1.1 (0.57)	1.46 (0.47)	0.37 0.20, 0.53	<0.001
2 min walk test (m)	Intervention	99.61 (81.01)	133.33 (47.67)	33.72 9.11, 58.33	0.010
	Usual care	113.50 (60.99)	152.59 (48.69)	39.07 21.05, 57.09	<0.001
**Outcome**	**Group**	**Baseline**	**12-week retest**	**Median difference**	**Wilcoxon signed rank test**
		**Median [IQR]**	**Median [IQR])**		***Z*,*p***
SwePASS-NV	Intervention	32 [8.50]	34 [6]	2	−3.28, 0.001
	Usual care	31 [4.75]	34 [4.5]	3	−3.34, <0.001
EQ5D index	Intervention	0.69 [0.40]	0.71 [0.20]	0.02	−1.33, 0.18
	Usual Care	0.72 [0.758]	0.82 [0.18]	0.10	−3.55, <0.001

For the secondary outcome measures, the regression model and Mann-Whitney U test showed no significant differences between groups at 12-week retest (Tables 3, 5), except for EQ5D-3L-scores where group allocation significantly predicted 12-week retest scores in favor of the usual care group (*p* = 0.003) (Table 3). There were significant within-group changes in both groups on MiniBesTest (*p* < 0.001), 10 MWT at preferred pace (intervention group: *p* = 0.007, usual care group *p* < 0.001), SwePASS-NV (Intervention group: *p* = 0.001, usual care group *p* < 0.001) and 2 MWT (intervention group: *p* = 0.01, usual care group *p* ≤ 0.001). Only the usual care group showed significant improvements in 10 MWT fast pace (*p* < 0.001) and EQ5D (*p* < 0.001) at 12-week retest when compared to baseline (Table 4). Within-group changes for the force-platform data were significant in favor of the intervention group in the domain of COP*_x_* with eyes open (*p* = 0.05) and COP*_y_* with eyes open (*p* = 0.01) and eyes closed (*p* = 0.03) (Table 5).

**Table 5:** Force platform data. Within- and between-group changes.

Outcome	Group	Baseline	12-week retest	Wilcoxon signed rank test	Mann-Whitney *U* test	
Force platform COP displacements (cm)		median [IQR]	median [IQR]	*Z*, *p*	*U*	*p*
RMS COPx eyes open	Intervention	1.32 [1.77]	1.28 [1.25]	0.05	280	0.37
	Usual care	1.25 [1.13]	1.39 [1.51]	0.72		
RMS COPy eyes open	Intervention	3.51 [3.16]	2.24 [2.25]	0.01	282	0.26
	Usual care	2.61 [2.83]	2.98 [2.46]	0.38		
RMS COPx eyes closed	Intervention	1.27 [1.34]	1.01 [1.09]	0.32	282	0.51
	Usual care	1.13 [1.33]	1.51 [1.20]	0.57		
RMS COPy eyes closed	Intervention	2.73 [1.72]	2.42 [2.05]	0.03	271	0.68
	Usual care	2.51 [1.44]	2.25 [2.33]	0.84		

**Figure 5:**
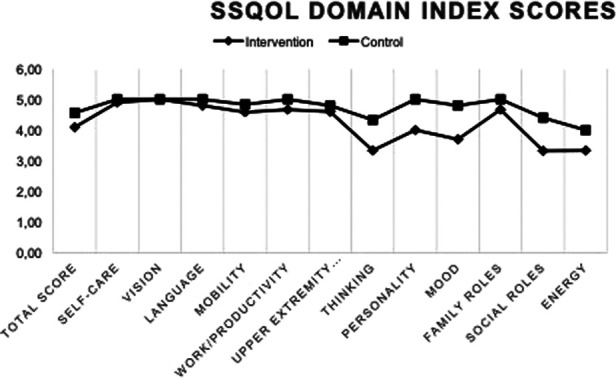
SSQOL-scores.

Regarding the SSQOL, 43 forms were returned and 17 of these had missing data. Two were discarded due to two missing items in a three-question domain. Both groups shared similar trends with regards to which domains had the highest (“vision” and “self-care”) or lowest (“energy”) scores. The usual care group had higher median scores at 12 weeks in all domains, but “vision” where scores were equal (Table 6) and had a higher total index score at 12 weeks post stroke. Differences between groups were more pronounced in the cognitive-social-mental components than in the physical health components of the SSQOL (Figure 5). Mann-Whitney U test showed significant group differences in index scores, all favoring of the usual care group in the domains of “language” (*p* = 0.005), “mobility” (*p* = 0.036), “upper extremity function” (*p* = 0.011), thinking (*p* = 0.011), personality (*p* = 0.019) and mood (*p* = 0.006) domains.

**Table 6:** SSQOL index scores.

Domain	Intervention group index score (*n* = 18)	Usual Care group index score (*n* = 23)	Mann-Whitney *U* test	
	Median [IQR]	Median [IQR]	*U*	*p*
Self-Care	4.90 [0.55]	5.00 [0.20]	256.00	0.15
Vision	5.00 [1.00]	5.00 [0.33]	210.50	0.91
Language	4.80 [0.60]	5.00 [0.20]	306.00	0.005
Mobility	4.58 [0.58]	4.83 [0.67]	285.50	0.04
Work/productivity	4.67 [1.00]	5.00 [1.00]	226.00	0.59
Upper extremity function	4.60 [1.25]	4.80 [0.20]	300.50	0.01
Thinking	3.33 [2.50]	4.33 [1.67]	266.50	0.11
Personality	4.00 [2.17]	5.00 [0.33]	292.50	0.02
Mood	3.70 [1.85]	4.80 [0.80]	308.50	0.006
Family roles	4.67 [2.08]	5.00 [1.00]	253.00	0.20
Social roles	3.33 [2.14]	4.40 [1.80]	263.00	0.14
Energy	3.33 [2.67]	4.00 [2.33]	243.00	0.34
Total score	4.09 [1.43]	4.56 [0.51]	281.00	0.05

The calculation of average number of weeks in physical therapy was based on the returned forms from the physical therapists (Supplementary Material). Participants in the intervention group: completed on average 7.94 (SD 3.45) weeks of physiotherapy. In the usual care group, the participants completed an average of 10.36 (SD 2.31) weeks of physiotherapy. Differences in how the forms were filled out made it difficult to determine the number of sessions completed by each participant.

## Discussion

Results show that there were no significant differences between groups following 12 weeks of intensive physiotherapy training with either I-CoreDIST or usual care when adjusted for baseline differences, suggesting that there were no added benefits from the implementation of I-CoreDIST during the sub-acute stage after a stroke. Our results are in line with previous research in stroke rehabilitation where results of clinical trials often are neutral (53, 54), meaning there is no statistical significant difference between groups at endpoint (55). We did encounter some well-known challenges in stroke rehabilitation RCT's, such as issues with recruitment rates, group heterogeneity and implementation fidelity that that are likely to have impacted upon results (53). In addition, the I-CoreDIST intervention is complex, defined as “containing several interacting components, targeting more than one organizational level of health care and offering considerable flexibility/tailoring” (56, 57). The intervention is low-cost and designed for implementation in clinical practice. While its flexibility allows for broad use and individualization, it is in opposition to the often highly standardized delivery of interventions in an RCT and would require increased power to yield statistically significant results. The registrations of content in treatment also suggest a degree of similarities between interventions as in the returned forms 71.4% of the usual care group reported having included postural control (Supplementary Material). However, interviews with a subgroup of participants (*n* = 19) revealed that experiences with participation in the study differed predominantly with regards to the content of therapy (58). Interviews confirm a greater focus on postural and movement control in the intervention group while participants in the usual care group describe an approach of structured training of strength and endurance measured through increased resistance or number of repetition (58).

Following 12 weeks of 3–5 weekly physiotherapy sessions, both groups showed both statistically and clinically significant improvements in measures of postural control and balance, sustained low levels of physical activity, and variable improvements in gait speed and distance.

For the primary outcomes, participants in both groups had a mean change near the previously reported MDC for TIS-modNV (29), indicating a true measure of improved trunk control exceeding that is associated with error. Only the usual care group showed statistically significant changes in activity levels for the categories of moderate activity and steps, equaling 56 moderate active mins/week and a daily average of 3,327 steps. Despite improvements in balance and that all participants had regained ambulation at 12-week retest, activity levels in both groups are well under the 150–300 min of moderate activity recommended for the general population in Norway (59) and the 20–60 min of aerobic activity 2–3 times/week recommended for the stroke population (60). There was a non-significant reduction in sedative minutes/day (Intervention: −27, Usual care: −7) and an increase in minutes of light activity/day (Intervention: 36, Usual care: 7) in favor of the intervention group. The high levels of sedative time, complete lack of vigorous physical activity and low average number of steps across groups is a cause for concern, both with regards to recovery and secondary prevention (60). Our results are in line with previous research on the stroke population (4), and may suggest suboptimal intensity in or duration of physical therapy sessions at baseline and little uptake of physical activity after the 12-week treatment period and retest. Apart from physical barriers, social factors, support and cognitive impairments have been suggested to influence levels of physical activity after a stroke (61, 62). These issues need further investigation.

With regards to secondary outcomes, improvements in PASS were statistically significant in both groups, though only the usual care group reached the MDC of 2.2 points. Both groups were within the category “good postural control” (31–36 points) at baseline and the previously reported ceiling effect in this measure (34, 63, 64). Both groups exceeded the required change of 5 points constituting substantial clinically important changes on the MiniBESTest (36), that together with improvements in TIS-modNV and PASS suggest overall improved postural control and balance in both groups. Force plate assessments of standing balance with eyes open and eyes closed showed statistically significant reduction in sway amplitudes in both AP and ML directions for the intervention group only implying improved balance control (49). This indicates that the focus on core activation and trunk control as recommended in the literature (13, 15, 16) and implemented in the I-CoreDIST intervention has resulted in reduced postural sway, that generally indicates improved postural stability (49, 65).

In measures of gait speed and distance, both groups exceeded the MCID on 10 MWT preferred pace (41) and fast pace (42), and displayed gait speeds well beyond the <0.8 m/s required for efficient community ambulation (66) at 12-week retest. Only the usual care group reached statistically significant within-group changes in 10 MWT fast pace. This suggests that the I-CoreDIST intervention did not target high walking speeds sufficiently.

Improvements in EQ5D were significant for the usual care group only and SSQOL-scores were generally lower in the intervention group. Group differences in SSQOL were more pronounced in the domains of thinking, personality, mood, social roles and energy than in the domains of self-care, vision, language, mobility, work/productivity and upper extremity function. The SSQOL and EQ5D indicate a lower HRQOL in the intervention group that seems more related to cognitive/mental than physical components. This may suggest a larger proportion of cognitive/mental problems in this group, which may have been caused by the stroke, result from the lower premorbid function, a higher age and stroke severity, or a combination of these. Exercise interventions are known to have small to moderate beneficial effects on HRQOL in physical and mental health domains that diminish at longer-term follow up, and no significant effects on societal or participatory domains, (67). The limited uptake of physical activity after the intervention, as indicated by the activity monitoring at 12-weeks along with lower HRQOL-scores on cognitive/mental components, supports these notions.

### Limitations

The major limitation of this study is that it is underpowered (*n* = 60) with regards to the sample size calculations (*n* = 74). In addition, ten participants were lost to follow-up in the intervention group. Four were excluded, and six discontinued physiotherapy or did not attend retest. The reasons given were mainly related to travel time to the physiotherapist/hospital and fear of Covid-19 on public transportation/in the physiotherapy clinic/hospital. With regards to implementation fidelity, further investigations into issues of recruitment and retention, such as barriers and effects of participation for both participants and physiotherapists and the quality of I-CoreDIST training and materials would have been beneficial. Participants in the usual care group, on average received physiotherapy for 2.4 weeks more than those in the intervention group. Registration forms revealed a vulnerability regarding absence, sick leave etc., particularly for the physiotherapists treating the intervention group. Only 1–2 physiotherapists had I-CoreDIST training on most sites, resulting in limited ability for another therapist to cover in case of absence. No additional training was required to treat the usual care group. These issues were further reinforced by Covid regulations and reallocation of staff related to the handling of pandemic. The 12-week follow up period is relatively short and a long-term follow up would have been beneficial.

## Conclusion

A 12-week physiotherapy program with either I-CoreDIST or usual care implemented during the first 12 weeks showed no differences between groups, except for significant gains in HRQOL in favor of the usual care group. Both groups showed significant improvements on measures of postural control, balance and gait.

## Data Availability

The datasets presented in this article are not readily available as we do not have permission from the ethical committee to share data. Requests to access the datasets should be directed to marianne.sivertsen2@nordlandssykehuset.no.

## Footnotes

^1^I-CoreDIST: I = individualised, Core = trunk, D = dual task, I = intensive, S = specific, stability, somatosensory stimulation, T = teaching, training. Individualised Core activation combined with DISTal functional movement
