# Supplementary material for: Effect of innovative vs. usual care physical therapy in subacute rehabilitation after stroke. A multicenter randomized controlled trial
Source: Front Rehabil Sci. 2022 Sep 19;3:987601. doi: 10.3389/fresc.2022.987601 (PMC9673903; doi:10.3389/fresc.2022.987601)
Supplement: Supplementary file 1 [file Datasheet1.pdf]

Supplementary material:

1. **Contents of physiotherapy in the usual care group**

Content of physiotherapy sessions	Number of participants where activity was part of physiotherapy, n (%)<br>N=14	Activity was part of physiotherapy treatment for number of weeks during 12-week follow-up,<br>median [IQR]
**Balance training**	13.0 (92.9)	9.0 [6.0]
**Strength training**	13.0 (92.9)	9.0 [7.0]
**Gait**	10.0 (71.4)	8.5 [12.0]
**Passive movements**	2.0 (14.3)	0.0 [0.0]
**Soft tissue mobilisations**	5.0 (35.7)	0.0 [2.0]
**Active assisted movements**	7.0 (50.0)	0.5 [2.0]
**Endurance training**	14.0 (100.0)	9.0 [5.0]
**Functional training**	9.0 (64.3.)	2.5 [12.0]
**Stretching**	4.0 (28.6)	0.0 [2.0]
**Heat**	1.0 (7.1)	0.0 [0.0]
**Postural control**	10.0 (71.4)	4.0 [12.0]
**Independent exercises in gym**	12.0 (85.7)	8.0 [8.0]
**Specific somatosensory stimulation**	9.0 (64.3)	2.5 [6.0]
**Pilates**	0.0 (0.0)	0.0 [0.0]
**Yoga**	1.0 (7.1)	0.0 [0.0]
**Sling-exercises**	0.0 (0.0)	0.0 [0.0]
**Outdoor activities**	9.0 (64.3)	2.0 [8.0]

2. **Content of physioterapy, I-CoreDIST group**

Exercises	Number of participants where activity/Exercise was part of physiotherapy n (%). N=16	Activity was part of physiotherapy treatment for number of weeks during 12-week follow-up, median [IQR]
**A: Sensori- and oculomotor function**		
**Sensorimotor activation and mobility of the foot and ankle**	15.0 (93.8)	5.5 [7.0]
**Sensorimotor activation and mobility of the hands**	12.0 (75.0)	1.0 [4.0]
**Sensorimotor activation of the face**	0.0 (0.0)	0.0 [0.0]
**Oculomotor exercises**	6.0 (37.5)	0.0 [2.0]
**B: Supine**		
**Bent banana-straight banana**	8.0 (50.0)	0.5 [3.0]
**The yes**	2.0 (12.5)	0.0 [0.0]
**The spider**	6.0 (37.5)	0.0 [4.0]
**The bridge**	11.0 (68.8)	3.0 [5.0]
**The shrimp**	10.0 (62.5)	2.5 [6.0]
**Standing on the wall**	10.0 (62.5)	2.0 [4.0]
**Balancing on the wall**	6.0 (37.5)	0.0 [3.0]
**Walking on the wall**	10.0 (62.5)	2.5 [7.0]
**The crab-wiggle/ball play**	2.0 (12.5)	0.0 [0.0]
**Walking on air**	4.0 (25.0)	0.0 [1.0]
**Sit up**	8.0 (50.0)	1.0 [4.0]
**Sit up 2**	4.0 (25.0)	0.0 [2.0]

**C: Side-lying**		
**The stick**	3.0 (18.8)	0.0 [0.0]
**Reach for the stars**	2.0 (12.5)	0.0 [0.0]
**D: Prone**		
**The eagle**	3.0 (18.8)	0.0 [0.0]
**The cat**	6.0 (37.5)	0.0 [4.0]
**E: Sitting**		
**Slowly diving**	5.0 (31.3)	0.0 [2.0]
**Reach all over**	10.0 (62.5)	2.0 [4.0]
**Rolling the ball**	12.0 (75.0)	1.5 [4.0]
**Hands up**	6.0 (37.5)	0.0 [1.0]
**The butterfly**	3.0 (18.8)	0.0 [0.0]
**Nodding**	1.0 (6.3)	0.0 [0.0]
**The angel**	4.0 (25.0)	0.0 [1.0]
**Pluto**	1.0 (6.3)	0.0 [1.0]
**Pelvic walk**	8.0 (50.0)	0.5 [2.0]
**Stand up**	7.0 (43.8)	0.0 [3.0]
**All rise**	8.0 (50.0)	1.0 [2.0]
**High sit to stand**	5.0 (31.3)	0.0 [3.0]
**F: Standing**		
**High kneeling**	4.0 (25.0)	0.0 [0.0]
**Squats**	15.0 (93.8)	5.5 [6.0]
**The wiggle no 1**	7.0 (43.8)	0.0 [4.0]
**The wiggle no 2**	9.0 (56.3)	1.5 [6.0]
**Calf-rise**	10.0 (62.5)	1.5 [4.0]
**The corner**	6.0 (37.5)	0.0 [2.0]

**The march**	14.0 (87.5)	5.0 [5.0]
**Play ball**	7.0 (43.8)	0.0 [2.0]
**The waiter**	5.0 (31.3)	0.0 [1.0]
**The bounce**	7.0 (43.8)	0.5 [3.0]
**G: Stepping and walking**		
**Stepping**	8.0 (50)	0.5 [4.0]
**Walking the ball**	3.0 (18.8)	0.0 [0.0]
**Push the therapist**	6.0 (37.5)	0.0 [1.0]
**Guided walking**	10.0 (62.5)	1.5 [7.0]
**Run away**	4.0 (25.0)	0.0 [1.0]
**Stairway to heaven**	11.0 (68.8)	3.0 [5.0]
